# Cardiovascular System during SARS-CoV-2 Infection

**DOI:** 10.3390/ijerph19031184

**Published:** 2022-01-21

**Authors:** Maciej Koźlik, Adrianna Błahuszewska, Maciej Kaźmierski

**Affiliations:** 1 Department of Cardiology and Structural Heart Disease, Medical University of Silesia, 40-635 Katowice, Poland; kazmierski.maciej@gmail.com; 2 Department of Rheumatology and Internal Medicine, Medical University of Bialystok, 15-276 Bialystok, Poland; reum@umb.edu.pl

**Keywords:** coronavirus disease 2019, cardiac injury, troponin, cardiac complications, prognosis

## Abstract

SARS-CoV-2 virus can not only damage the respiratory system but may also pose a threat to other organs, such as the heart or vessels. This review focuses on cardiovascular complications of COVID-19, including acute cardiac injury, arrhythmias, biomarkers, accompanying comorbidities and outcomes in patients diagnosed with SARS-CoV-2 infection. The research was conducted on the databases: PubMed, Springer, ScienceDirect, UpToDate, Oxford Academic, Wiley Online Library, ClinicalKey. Fifty-six publications from 1 November 2020 till 15 August 2021 were included in this study. The results show that cardiac injury is present in about 1 in 4 patients with COVID-19 disease, and it is an independent risk factor, which multiplies the death rate several times in comparison to infected patients without myocardial injury. New-onset cardiac injury occurs in nearly every 10th patient of the COVID-19-suffering population. Comorbidities (such as hypertension, cardiovascular disease and diabetes) severely deteriorate the outcome. Therefore, patients with SARS-CoV-2 infection should be carefully assessed in terms of cardiac medical history and possible cardiological complications.

## 1. Introduction

After almost two years of global epidemics, we are certain that complications of COVID-19 do exist and have a tremendous impact on our psychical and mental condition [1,2]. SARS-CoV-2 has emerged as a life-threatening danger that damages the respiratory system resulting in pneumonia or acute respiratory distress syndrome (ARDS) [3], as well as the nervous system, causing headache, weakness, smell or taste loss [4,5]. In the vast majority of infected patients, the virus presents with mild symptoms, and a minority have a severe course of COVID-19, requiring hospitalisation or intensive care [6]. Aside from the respiratory and nervous systems, the cardiovascular system may be affected as well regarding short and long-term complications. The long-term results presumably will be subjected to further observation in the years following the epidemic. Thus, it is crucial to prepare for the challenges ahead and direct our research efforts to improve health care quality.

This review highlights possible cardiological complications in patients with SARS-CoV-2 infection and presents the outcome of patients with cardiovascular risk factors to illustrate what can be possibly encountered and should be expected during hospitalisation or ambulatory treatment.

## 2. Materials and Methods

**Figure 1:**
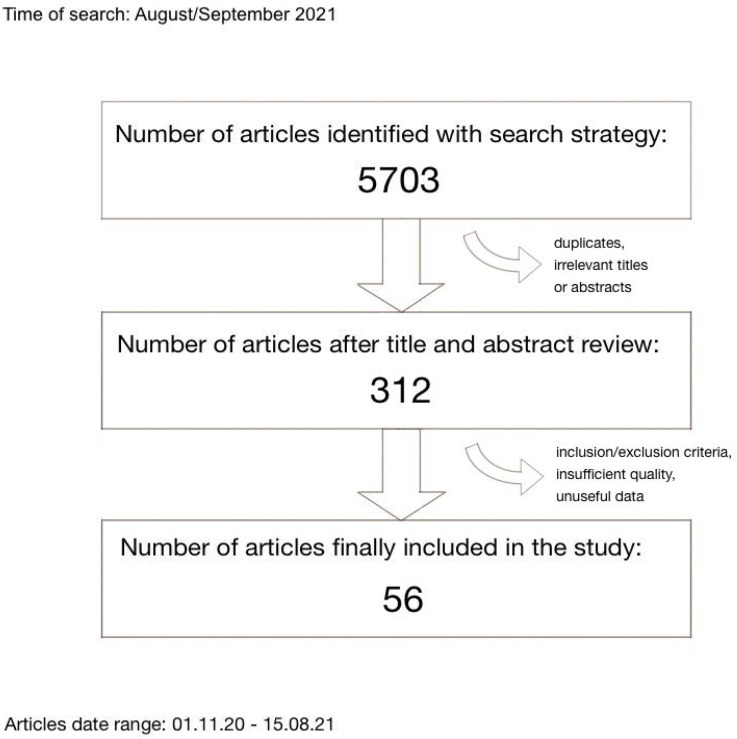
Study selection.

**Figure 2:**
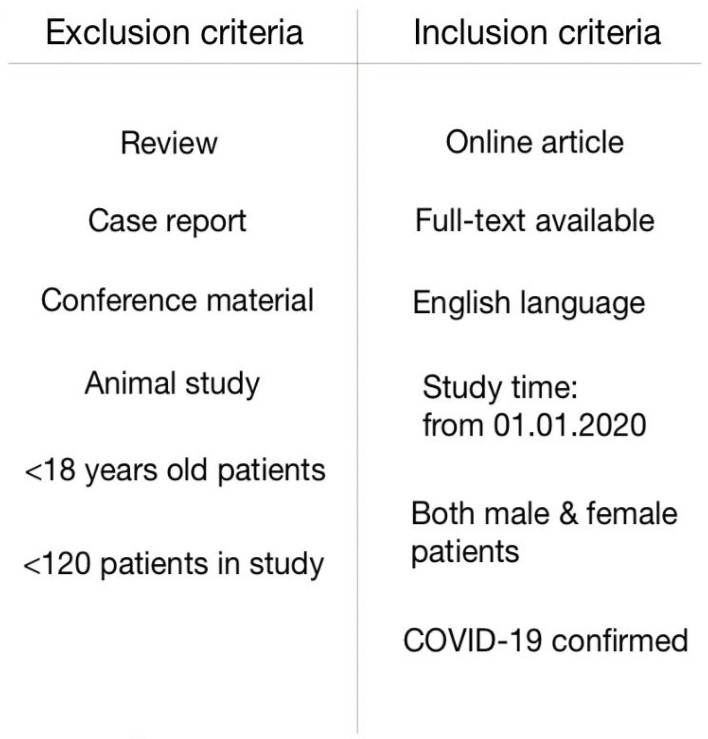
Exclusion and inclusion criteria.

There were three reviewers working independently who performed the study selection, which included: searching for articles, title and abstract screening, full-text reading and final article selection. It was based on study characteristics (title, date of publication, country, language, study time period, citation amount, work type), study design (inclusion and exclusion criteria, healthcare setting, examination and data collecting process), population characteristics (sample size, age, whether patients had COVID-19 disease confirmed, demographic characteristic, past medical history, illness severity), connections, correlations or links in patient population (between cardiac injury, myocardial infarction, myocarditis, arrhythmias, hypertension, diabetes, cardiovascular artery disease and SARS-CoV-2 infection) and article quality (quality of reporting, language, intervention generalisability, choice of outcome measure and level of details that determined the final results in the article). Specifically, we included studies that evaluated patients with confirmed COVID-19 disease and cardiac injury (based on troponin level). All reviewers assessed the risk of bias of included studies independently. Discrepancies were resolved by mutual agreement and consensus. We have searched PubMed (majority), Springer, ScienceDirect, UpToDate, Oxford Academic, Wiley Online Library and ClinicalKey databases using the following terms: “cardiac complications COVID-19”, “cardiac complications SARS-CoV-2”, “cardiological complications COVID-19”, “cardiological complications SARS-CoV-2”, “cardiac injury COVID-19”, “cardiac injury SARS-CoV-2”, “myocardial infarction COVID-19”, “myocardial infarction SARS-CoV-2”, “acute cardiac syndrome COVID-19”, “acute cardiac syndrome SARS-CoV-2”, “troponin COVID-19”, “troponin SARS-CoV-2”, “myocarditis COVID-19”, “myocarditis SARS-CoV-2”, “arrhythmia COVID-19”, “arrhythmia SARS-CoV-2”, “atrial fibrillation COVID-19”, “atrial fibrillation SARS-CoV-2”, “ECG changes COVID-19”, “ECG changes SARS-CoV-2”, “biomarkers COVID-19”, “biomarkers SARS-CoV-2”, “comorbidities COVID-19”, “comorbidities SARS-CoV-2” and screened similar articles as well. We searched for original research articles in English from 01.11.2020 to 15.08.2021. Firstly, we excluded repeated articles. Afterwards we excluded irrelevant titles and out-dated articles (before 01 November 2020). Next, we excluded conference materials, other reviews, updates and errata, animal reports and case reports. Later we excluded studies with insufficient sample size (<120 patients), non-adult studies (<18 years old patients), only-male/only-female studies and with a study time before 01.01.2020. In the end, we performed an advanced quality assessment and included 56 articles (6 articles from 2020 year and 50 from 2021 year) in our literature review. Here we present a table (Table 1) including articles which were included in this review. Actions performed are visualised in Figure 1, exclusion and inclusion criteria are presented in Figure 2.

## 3. Results

### 3.1. Cardiac Injury

#### 3.1.1. Frequency and Characteristic

**Figure 3:**
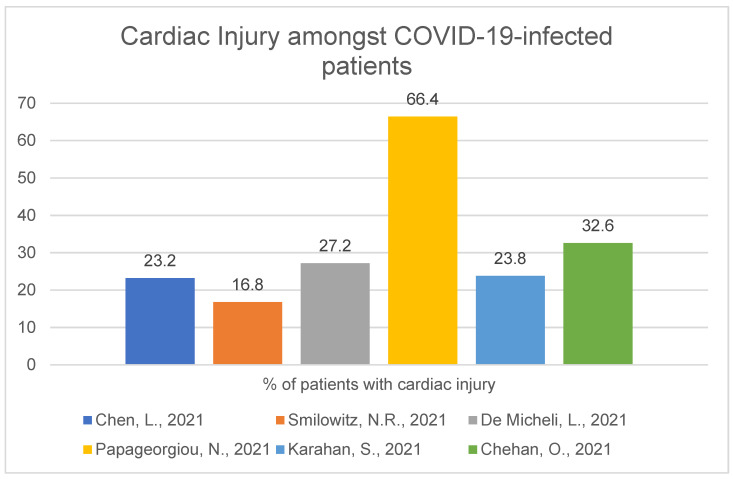
Cardiac injury amongst COVID-19-infected patients [7,8,9,10,11,12].

We used 15 articles in this section. Cardiac injury was present in 16.8% to 66.4% of subjects, and in the vast majority of reviewed articles, it was diagnosed based on elevated troponin level (cTn—cardiac troponin, hs-cTn—high sensitivity cardiac troponin) sometimes along with additional abnormality (for instance, new-onset abnormal ECG or echocardiography finding). A study in the group of 181 COVID-19-positive patients showed 23.2% of them had cardiac injury diagnosed on the basis of elevated biomarkers (high-sensitivity troponin I, myohemoglobin and creatine kinase myocardial (*p* < 0.001, >99th percentile)). The median age was 55 (IQR, 46–65) years [7]. A study of 2895 COVID-19-positive patients in New York University School of Medicine showed that cardiac injury was present in 16.8% of patients, and troponin levels (>99th percentile, adjusted for sex and age) were considered to be indicative of myocardial necrosis [8]. In the next study of 313 COVID-19-positive patients in Italy, hs-cTnI (high-sensitivity cardiac troponin I) elevation was present in 85 patients (27.2%). The mean age for the whole group was 66.1 (55.1–79.4) [9]. There is one multi-centre study with higher percentages than others, carried out in the United Kingdom, which included 434 COVID-19-positive patients (mean age 66 (56–80) years) and which demonstrated cardiac injury in 288 patients (66.4%). Median LVEF (left ventricle ejection fraction) was significantly lower for troponin(+) patients as compared to troponin(-) patients [10]. In research conducted in Istanbul, Turkey, 77 of 326 (23.8%) consecutive patients had a new acute cardiac injury (based on serum troponin I value >19.8 ng/mL, mean age 58.4 ± 15.2 years) [11]. In the last research of 270 COVID-19-positive patients, cardiac injury was present in 32.6% of infected patients during hospitalisation [12]. All six studies mentioned above can be seen in the figure below (Figure 3).

Critical or severe patients have higher levels of troponin during hospitalisation and are more likely to have a cardiac injury with the range from 28.3% to 51% [13,14,15,16]. In a study of 330 patients, cardiac injury (based on hs-cTnI levels) was diagnosed in 32.4% of patients (104), and the whole group consisted of only severe and critically ill COVID-19-positive patients [14]. In the multi-centre study of 2878 SARS-CoV-2-positive critical patients in France, troponin was elevated above each centre’s threshold in 32.4% and in 58.5% of the deceased group [16]. In the study of 243 intubated COVID-19-positive patients from five different hospitals, 51% had troponin levels above the upper limit [15]. Another paper of 201 SARS-CoV-2-positive patients with myocardial injury (based on cTnI) shows that troponin was elevated above the normal level of 0.04 ng/mL in mild patients and three times over the threshold (>0.12 ng/mL) in critical patients (28.3%, *p* < 0.001, critical patients were those who needed mechanical ventilation) [13]. In another article of 218 patients with myocardial injury, there were also major differences in troponin elevation between critical and mild COVID-19-positive patients (28.8% critical vs. 4.8% non-critical, *p* < 0.0001) [17].

**Figure 4:**
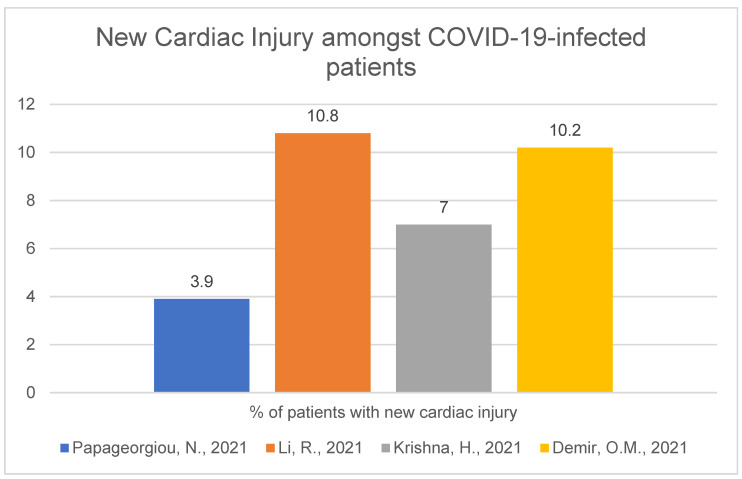
New cardiac injury amongst COVID-19-infected patients [10,17,18,19].

The situation is different when speaking of first-detected cardiac injury. Here the numbers are lower with a range: 2.9–10.8%. A Chinese study has revealed that hs-cTnl (high-sensitivity cardiac troponin type I) was elevated in COVID-19-positive patients in 10.8% of 218 enrolled patients (mean age was 62 (IQR: 55, 69) years). These patients presented without previous cardiovascular symptoms or past medical history involving cardiovascular disease and were admitted to the hospital because of typical SARS-CoV-2 symptoms [17]. In the American study of 179 patients (mean age 59.8 ± 16.9 years), myocardial injury was present in only 7% of patients (significant troponin level elevation accompanied by new ventricular dysfunction or electrocardiographic abnormalities) [18]. Scientists from the King’s College of London conducted a randomised study of 172 SARS-CoV-2-positive patients (mean age 55.1 ± 13.9 years), which showed that 10.2% developed a major cardiac injury (peak hs-cTnT >20× ULN) [19]. In the previously presented analysis, new cardiac abnormalities occurred in 3.9% of 434 COVID-19-positive patients [10]. Concerning athletes, a study showed that only 2.9% had abnormal newly-detected cTnI levels with no serious symptoms (laboratory 99th percentile, 0.035 ng/mL.). All these athletes were referred for CMR. No athlete had abnormal findings detected by CMR: no ventricular dysfunction, delayed myocardial enhancement, abnormal T2 weighted imaging or pericardial pathology. Limitations of this result could be that the authors did not use hs-cTnI [20]. The four previously mentioned studies [10,17,18,19] are assembled in the figure below (Figure 4). The information from [20] is not included in the figure because it concerns athletes.

Critical or severe patients have higher levels of troponin during hospitalisation. In Wuhan, 40 (30.3%) consecutive patients displayed acute cardiac injury (based on hs-TnI above the 99th-percentile upper reference limit, mean age 61 ± 13 years) [21]. These could be due to more critical cases (62.1% severe or critical).

#### 3.1.2. Hospitalisation and Outcome

We used 23 articles in this section. Cardiac injury was a crucial and independent risk factor for mortality in COVID-19-infected patients [12,21,22,23] and mortality was usually higher in the cardiac injury group [7,9,23,24,25]. Patients with cardiac injury were older [7,11,22,26], had more comorbidities [22,25,26], were mostly male [10,22], had more serious disease [7,22,23] or were more likely to require mechanical ventilation [21,22]. Age is an independent predictor of myocardial injury in COVID-19-positive patients [10]. Critical patients with myocardial injury (serum troponin I > 99th percentile ULN) are more likely to die [11] (17 times more likely) or develop ARDS [11] (11.5 times more likely) than those without myocardial injury. Critical patients with higher troponin levels (>10 ULN) were more likely [15] (2.7 times more likely) to die than those with any troponin elevation (< ULN). Critical patients with high hs-cTnI (>28 pg/mL) levels were more likely to die [27] (2.637 times more likely, 95% CI, 1.058-6.570, *p* = 0.037)) than those with low levels, as well, hs-cTnI was an independent risk factor of mortality [27] in these patients, which indicates that hs-cTnI levels may be useful in assessing mortality risk in critically ill patients with COVID-19. Elevation of cardiac biomarkers is an independent predictor of mortality [7,8,16,18,19,28,29,30,31] or severity of illness [10], even in patients with no previous history of CAD or heart insufficiency [16]. These are myoglobin [7], CK-MB [7] and troponin [8,10,18,19,28,29,30,31,32]. Length of stay at the hospital in days is shorter for patients with negative troponin levels [10]. There is a study of 196 COVID-19-positive patients that showed that cTnI (≥21 ng/L) is a better prognostic value for 30-day all-cause mortality in comparison with CRP, LDH and D-Dimer [31]. A previously presented study of 181 COVID-19-positive patients showed patients with cardiac injury were older and had more comorbidities (most common were hypertension, coronary heart disease and arrhythmias), and the cardiac injury group (42 patients) required noninvasive ventilation more often (20 (47.6%) vs. 14 (10.1%), *p* < 0.001), more often had severe disease (36 (85.7%) vs. 47 (33.8%), *p* < 0.001) and mortality was much higher in the cardiac injury group (22 (52.4%) vs. 12 (8.6%), *p* < 0.001) [7]. The situation is no different in another study where the COVID-19-positive group was associated with a more severe presentation of SARS-CoV-2-infection (critically severe, 26.0% vs. 7.2%, all *p* < 0.001). Compared with those without myocardial injury, more COVID-19-positive patients with myocardial injury required oxygen inhalation, non-invasive ventilation, invasive mechanical ventilation, antibiotic treatment and hemoperfusion therapy (all *p* < 0.001). Mortality rate was significantly higher in patients with myocardial injury (63.5% vs. 13.0%, *p* < 0.001) [22]. A study of 400 COVID-19-positive patients in this section show that those with cardiac injury (troponin >99^th^ percentile) were older (52.5 (42.8–68.0) vs. 49.0 (36.0–0.0) years), myocardial injury event was much higher in patients with comorbidities: hypertension (12/46 (26.1%) vs. 50/354 (14.1%)), hyperlipidemia (4/46 (8.7%) vs. 7/354 (2.0%)) and chronic kidney insufficiency (3/46 (6.5%) vs. 2/354 (0.6%)) [26]. In the study of 226 patients, the rate of myocardial injury and myocardial infarction were significantly higher among deceased patients as compared with those who recovered (54.4% vs. 20.3%, *p* < 0.001; 20.6% vs. 6.3%, *p* = 0.003) [24]. The study of 367 patients showed that patients with myocardial injury have more comorbidities than patients without myocardial injury (hypertension: 78% vs. 42%, diabetes 44% vs. 21%, coronary artery disease 17% vs. 5.1%). Patients with myocardial injury had a higher risk for short-term mortality than those without myocardial injury (20% vs. 12%, *p* < 0.0001; unadjusted HR 4.44, 95% CI 2.13–9.25, *p* < 0.001) and for major adverse events (35% vs. 11%, *p* < 0.0001; unadjusted OR 4.29, 95% 2.50–7.40, *p* < 0.0001) [25]. In another research of 324 COVID-19-positive patients, death occurred in 54.5% and 3.2% of the patients with and without myocardial injury, respectively. Notably, 75.3% of patients with myocardial injury and 6.5% without myocardial injury developed ARDS. The median serum troponin I levels were 306 (72–852) and 2.5 ng/mL (1.3–5.5) in the deceased and surviving patients, respectively (*p* < 0.001) [11]. In the group of 243 critical patients, mortality was listed at the level of 22.7% among patients with COVID-19 and troponin over the upper limit of normal value (0.04 ng/mL) and 61.5% for those COVID-19-positive and troponin levels >10 times the upper limit of normal value (*p* < 0.001) [15]. Hs-cTnI was an independent marker for a critical outcome in a group of 726 COVID-19-positive patients (>28 pg/mL) (OR, 2.899; 95% CI, 1.743–4.822, *p* < 0.0001) [8]. The study of 2873 SARS-CoV2-infected patients, indicated that nine variables were independent predictors for in-hospital mortality, including troponin (HR: 2.150; 95% CI: 1.155–4.001; *p* = 0.016) [28]. Next, research of 880 COVID-19-positive patients pointed out that initial hs-cTnT (high-sensitivity cardiac troponin type T) was associated with composite adverse outcomes with a median hs-cTnT of 11 ng/L in patients who were living and did not require mechanical ventilation versus 31 ng/L in patients who expired or required mechanical ventilation [33].

An interesting study showed that there is no association between the initial viral load (iVL) and cardiac injury (based on increased >100 ng/L hs-cTnI level); however, both iVL and cardiac injury were independent predictors of mortality [12].

#### 3.1.3. Myocardial Infarction

We used three articles in this section. Myocardial infarction in COVID-19-positive patients is not common [9,25,34]. A study on 346 patients with elevated hs-cTnT conducted in the UK has demonstrated that there were no COVID-19-positive patients in the group of 115 patients with Type 1 MI, and in the group of 231 patients with Type 2 MI/myocardial Injury there were 36 (16%) patients who were COVID-19-positive. Cardiac causes of MI were more common in patients without SARS-CoV-2 virus (positive: 11.1% vs. negative: 33.3%, *p* = 0.22), of which tachyarrhythmia and heart failure were the most likely mechanisms (15.7% and 15.1%) [34]. Next, a study on 313 patients of whom elevation was present in 85 patients (27.2%) and of those 85 patients, only 11.7% had criteria for MI: 7% had type 2 MI, and 4.7% had type 1 MI [9]. In another work of 367 patients, only 5% of patients with elevated troponin (169) were adjudicated as acute MI, with three patients classified as type 1 MI and five patients as type 2 MI [25].

#### 3.1.4. Comparison of the Situation before COVID-19 Pandemic and Now

We used three articles in this section. The articles show us that admission for acute cardiac syndrome decreased between 40.4% and 47% [35,36], the severity of cases increased between 1.2 and 2.5 times [35,36], admission time was prolonged between 21% and 22% [35], or there were more late presentations [37], mortality increased in COVID-period 3.4 times [35] compared to last year and cardiac complications after hospitalisation were between 2.8 and 4 times more frequent [35,37]. In Berlin, Germany, a study of 355 patients showed that although the admission for acute myocardial injury decreased (by 47%) during the pandemic, the mortality has risen significantly (5.2% to 17.7%, *p* < 0.05). Patients demographically did not differ comparing the pre-COVID-19 and COVID-19 periods; the cases were much more serious though (elevated troponin, lower LVEF (39 ± 16 in 2020 vs. 46 ± 16 in 2019, *p* < 0.05), higher demand for inotropic support). Admission time was also prolonged (22% for STEMI and 21% for NSTEMI). Cardiac complications developed much more often (17.6% vs. 6.3%), and the rehabilitation period was longer than in the pre-COVID-19 period [35]. In another paper from Japan, there were many more late presentations with STEMI compared with pre-COVID-19 times (25.4% vs. 14.2%, *p* = 0.03). Primary PCI was performed less often (68.3% vs. 82.5%, *p* = 0.009) and mechanical complications occurred much more often also (3.6% vs. 14.3%, *p* < 0.001) [37]. In Spain, the number of patients with acute cardiac syndrome decreased by 40.4% (2020 vs. 2019). A total of 11% of acute cardiac syndrome patients in 2020 were COVID-19-positive, and many more patients with SARS-CoV-2 virus (46.2%) had acute cardiac syndrome at admission compared to the COVID-19-negative patients (11.4%) in the group from 2020 (as a secondary diagnosis—SARS-CoV-2 infection was the primary). There were significantly more in-hospital patients in severe condition with acute cardiac injury in 2020 compared with a cohort from the same timeframe of 2019 (15.3% vs. 6.1%, *p* = 0.007). In the COVID-19-positive group of 118 patients (from 2020 with acute cardiac injury and SARS-CoV-2-positive) COVID-19-positivity was an independent mortality predictor, and the SARS-CoV-2 positive test was independently associated with 30-day mortality [36].

#### 3.1.5. Myocarditis

We used five articles in this section. Acute myocarditis occurred in some COVID-19 patients and was usually focal but not common [25,38]. One of the studies with the biggest number of patients (1160) showed that acute myocarditis in COVID-19 patients developed in only 1% of cases [38]. Pericardial effusion was seen more frequently [21] (9%), [39] (9%). Another work of 367 patients showed that myocarditis was rare with clinical suspicion in only three patients in whom there was no definite confirmatory testing with cardiac magnetic resonance imaging or biopsy [25].

In the population of athletes, myocarditis or pericarditis was not common. Only 0.4% of athletes had myocarditis after COVID-19 infection [40], and 0.26% had pericarditis [40]. In another study, 3% had myocarditis [41], and 1.5% had pericarditis [41], usually without symptoms.

The situation is different in severe and critical COVID-19 patients. In a UK study concerning 148 severe COVID-19-positive and troponin(+) patients who underwent CMR (cardiac magnetic resonance), pericardial effusion was seen in 8/148 (5%). A total of 47/148 patients had a non-ischaemic pattern of myocardial injury, and 40/148 patients had a myocarditis-pattern injury (including four with coexisting inducible ischaemia and three with coexisting myocardial infarction). In the group of patients with myocarditis-pattern injury, only 12 (8% from the whole population) had findings consistent with active myocarditis (8 with a regional elevation of both native T1 and T2, 4 with a regional elevation of T2 only) [39].

### 3.2. Arrhythmias

#### 3.2.1. Frequency and Characteristic

We used nine articles in this section. Arrhythmias, which occurred during hospitalisation, were present with a range between 7.2% and 26.5% of patients. Studies from China, Denmark, Portugal, two from the US and Germany showed the frequency of arrhythmias: 7.2% of 390 patients [42], 11.7% of 692 patients [43], 26.5% of 166 patients [44], 18.3% of 186 [45] and 10% of 310 [46] with the vast majority being atrial fibrillation [42,44,46,47,48]. A total of 17.6% of 9564 patients had AF during hospitalisation [47], and 8% of 1029 had atrial arrhythmia [48] (these two studies focus only on AF or AA, respectively).

The majority of abnormal arrhythmias were atrial arrhythmias: 89.3% (6.4% from the whole population of 390 patients) [42], 82% (7.4% from the whole population of 310 patients) [46] and 8% from the whole population of 1029 patients [48]. The most common was atrial fibrillation, with the range from 4.3% to 10.4% of COVID-19-positive patients in selected studies presenting with such percentages: 5.4% of 390 [42], 4.3% of 186 [45], 10.4% of 517 [49] and with the range from 62% to 75% amongst the population with arrhythmia in selected studies: 75% [42], 62% [44], 62% [45].

**Figure 5:**
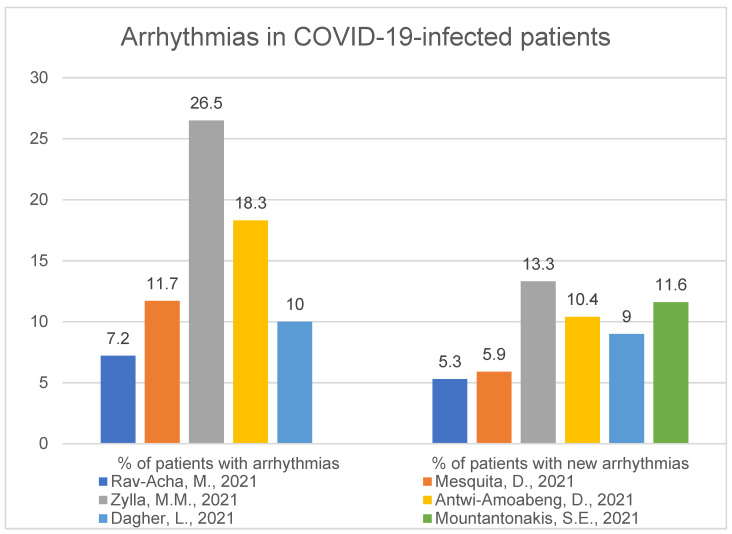
Arrhythmias and new arrhythmias in COVID-19-infected patients [42,43,44,45,46,47].

Completely new-onset arrhythmias happened not so rarely in 5.3% to 13.3% of patients. Presenting with such numbers were: 5.3% (76% AF) of the whole study population [42], 5.9% [43], 13.3% [44], 10.4% (95% AF) [45], 9% (82% atrial arrhythmias) [46] and 11.6% [47]. New-onset atrial arrhythmias in the study of 1029 people involved 4.5% of the whole group of patients [48]. There was a visible elevation in arrhythmia prevalence with increasing disease severity demonstrated in 28.6% of critical patients having new arrhythmia (*p* < 0.001) [42] and in another work, 25.2% of ICU patients [50]. The percentage of SARS-CoV-2-positive patients with both previous and new arrhythmias from previously selected studies [42,43,44,45,46,47] are presented in the next figure (Figure 5). Study [48] was not presented in the figure because it has taken only atrial arrhythmias into account.

#### 3.2.2. Hospitalisation and Outcome

We used eight articles in this section. Patients with new-onset arrhythmias were older [42,49,51] and presented more frequently with some comorbidities (hypertension, diabetes, cardiovascular artery disease or valvular heart disease) at admission [42,49,51] and stayed longer in hospital in comparison with the non-arrhythmia group [51]. Patients with arrhythmias were in general more likely to have some comorbidities [52]: hypertension (153 (38.6%) vs. 17 (25.4%); *p* = 0.04), coronary heart disease (21 (24.7%) vs. 29 (7.7%); *p* < 0.001) and diabetes mellitus (20 (23.5%) vs. 52 (13.8%); *p* = 0.025). In a study mentioned before of 517 patients, those with new arrhythmias were older (81.6 vs. 66.5 years old, *p* < 0.001) and more frequently presented hypertension (74% vs. 47%, *p* < 0.001), cardiomyopathy (9% vs. 1%, *p* = 0.002), previous heart failure admission (9% vs. 0.4%, *p* < 0.001), previous episodes of atrial fibrillation (83% vs. 1%, *p* < 0.001) and bigger left atrium (47.8 vs. 39.9 mm, *p* < 0.001) [49]. However, there was one study of 1029 COVID-19-positive patients that demonstrated that patients with new-onset arrhythmias were significantly younger compared to patients with previous arrhythmia or without, and also had higher BMIs (*p* < 0.05). Patients with new-onset atrial arrhythmia less often had a history of heart failure and coronary artery disease compared with recurrent and chronic persistent atrial arrhythmias patients (*p* < 0.05) [48]. More critical COVID-19-positive patients developed more arrhythmias compared to patients with mild or without symptoms [13,42,53,54], whilst in one of the studies, 28.6% of patients (critical) and 17.9% of patients (severe) presented with arrhythmia in the new arrhythmia group compared with 7.2% (critical) and 8.8% (severe) in the non-arrhythmia group (*p* <0.001) [42]. In a study of 463 COVID-19-positive patients, the proportion of critically ill patients was higher among patients with arrhythmias than those without arrhythmias (38 (44.7%) vs. 80 (21.2%); *p* < 0.001), suggesting an association between arrhythmias and adverse COVID-19 outcomes, and the all-cause mortality rate was higher in patients with arrhythmias than in those without arrhythmias (22 (25.9%) vs. 38 (10.1%); *p* < 0.001). Survival analyses showed that arrhythmias were associated with a high mortality rate (*p* < 0.001) [52]. In Bologna, Italy, 106/216 COVID-19-positive patients had abnormal ECG at the beginning of hospitalisation and increased troponin values occurred more often in patients who developed major adverse events (*p* = 0.04 and *p* = 0.02). Concerning ECGs after 7 days (159), abnormal findings were reported in 53.5% of patients, and they were more frequent in those with major adverse events (MAE, *p* = 0.001). The multivariable analysis showed the presence of abnormal ECG at 7 days of hospitalisation was an independent prediction factor of a major adverse event (HR 3.2; 95% CI 1.2–8.7; *p* = 0.02) [53].

### 3.3. Accompanying Comorbidities

We used twenty-nine articles in this section. COVID-19-positive patients with more comorbidities are associated with a more severe duration of illness [13], more often require intensive care [55] or are at a higher risk of death [11,16,24,56]. Male sex is prevalent among patients who died during hospitalisation [23,24,27,57,58] and is associated with higher mortality [23,56]. Age is an independent predictor of higher mortality in COVID-19-positive patients [16,55,56,59,60], increasing the risk of intensive care necessity [55] or illness severity [27]. Cardiovascular artery disease [55,56], diabetes mellitus and hypertension [55] are associated with higher mortality amongst COVID-19-positive patients and associated with intensive care or mechanical ventilation [55]. Critical patients are more likely to have the underlying cardiac disease [23], more comorbidities [13], to be male [13,23] and older [13,23,54]. About one-quarter of patients with heart failure die when they get infected with the SARS-CoV-2 virus [57]. There was a large study of 1,212,153 patients with a history of HF (heart failure), 8383 of which were COVID-19-positive (The Premier Healthcare Database), which concluded that patients with HF hospitalised with COVID-19 are at high risk for complications, with 24.2% dying during hospitalisation. Those who suffered from COVID-19 and had a past history of HF were more likely to have post-hospitalisation care (41% vs. 13%) or were referred to hospice (6.7% vs. 4.1%) than COVID-19-negative patients with HF. Patients with a previous history of HF and COVID-19 had significantly greater in-hospital resource use (mechanical ventilation, central venous catheter insertion) compared with patients hospitalised with acute HF and without COVID-19 (Intensive Care Unit care 29% vs. 15%, mechanical ventilation 17% vs. 6%, venous catheter 19% vs. 7%) Mortality amongst COVID-19 patients was greater in patients with acute heart failure 24.2% vs. 2.6% [57].

**Figure 6:**
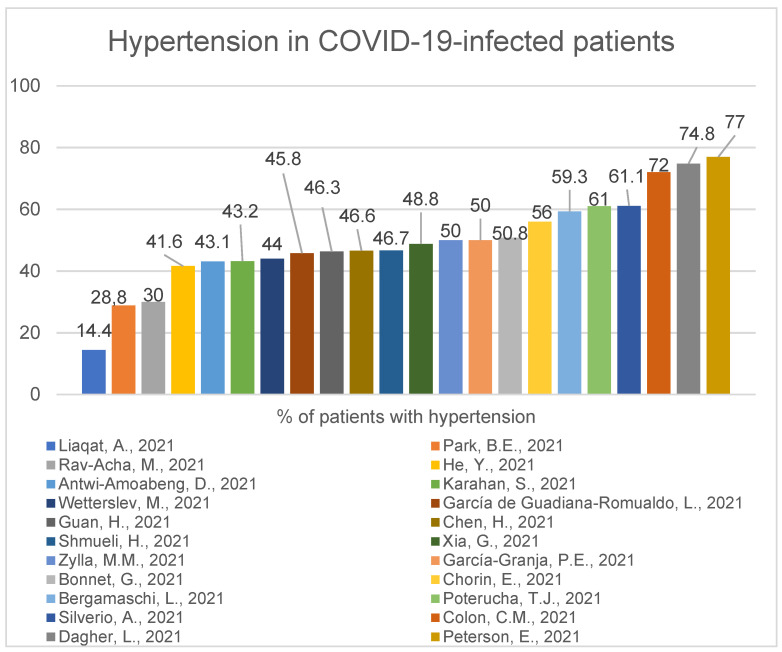
Hypertension in COVID-19-infected patients (median—47.8) [11,13,14,16,24,27,28,30,33,42,44,45,46,49,50,52,53,55,56,60,61,62].

**Figure 7:**
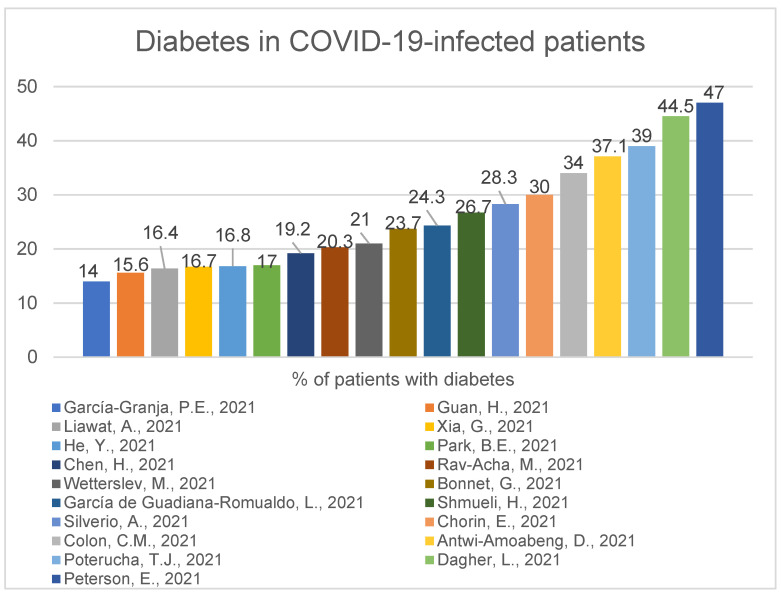
Diabetes in COVID-19-infected patients (23.7—median) [13,14,16,24,27,28,30,33,42,45,46,49,50,52,55,56,60,61,62].

**Figure 8:**
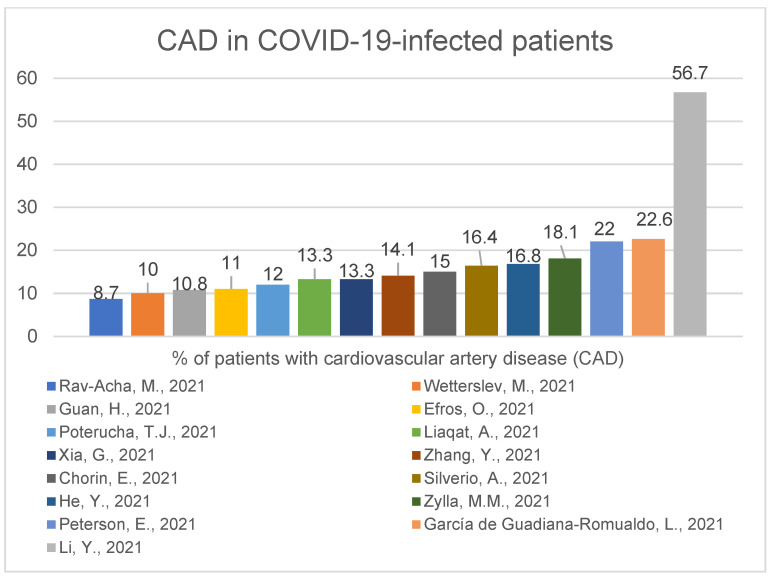
CAD in COVID-19-infected patients (14.1—median) [13,14,23,24,28,30,32,33,42,44,50,52,56,58,60].

A study of 324 COVID-19-positive patients shows us that hypertension and chronic kidney disease were significantly more common among the deceased compared with surviving patients. Myocardial injury was present in 84% of the deceased group, whereas 12.8% of the surviving patients had a myocardial injury (*p* < 0.001) [11]. One interesting work of 320 COVID-19-positive patients showed that predictors for elevated troponin levels were age (odds ratio (OR), 1.04; 95% confidence interval (CI), 1.01-1.06), female sex (OR, 3.03; 95% CI 1.54–6.25), low systolic blood pressure (OR, 5.91; 95% CI 2.42–14.44) and increased creatinine level (OR, 2.88; 95% CI 1.44–5.73) [32]. A study of 2878 COVID-19-positive hospitalised patients conducted in France showed 72.6% had at least one, 41.6% had at least two and 19.9% had at least three cardiovascular risk factors, as well, it showed that male sex (HR 1.69, 95% CI 1.11–2.57; *p* = 0.01), older age (hazard ratio (HR) 1.05, 95% confidence interval (CI) 1.03–1.06; *p* < 0.001), diabetes (HR 1.72, 95% CI 1.12–2.63; *p* = 0.01), chronic kidney failure (HR 1.57, 95% CI 1.02–2.41; *p* = 0.04) and elevated troponin (HR 1.66, 95% CI 1.11–2.49; *p* = 0.01) were independently associated with in-hospital death [16]. In the next study, 29% of 1169 COVID-19-positive patients in Italy had a previous history of some cardiac disease. Of these 29%, 60% had hypertension, 40% heart failure, 35% coronary artery disease and 25% atrial fibrillation. Only 18% had no comorbidities [38]. In a group of 1034 COVID-19-positive patients, these with some cardiovascular problems (who according to American Society of Echocardiography needed trans-thoracic echocardiogram) were more often admitted to ICU than those without any implications for examination (58.3% vs 18.9%) [61]. A study of 355 individuals demonstrated that patients with cardiovascular artery disease are at significantly higher risk of inpatient death than patients without cardiovascular artery disease (31% vs. 20%, *p* = 0.046), also age is significantly associated with higher mortality (1.041, 95% CI: 1.017–1.066, *p* = 0.001) in COVID-19 infected patients [56]. Another previously presented work of 226 patients demonstrated male sex was significantly dominant in patients who died during hospital stay compared to those who recovered (75% vs. 57%, *p* = 0.016), and thirty-six patients underwent percutaneous coronary intervention, which was significantly prevalent in the deceased cohort (11.8% vs. 3.2%, *p* = 0.025) [24]. In Korea, a study of 2269 COVID-19-positive patients demonstrated that diabetes mellitus (*p* < 0.001) and hypertension (*p* < 0.001) were associated with the increased requirement of intensive care and invasive MV (mechanical ventilation) and in-hospital death. Coronary artery disease (22.2% vs. 2.7%; *p* < 0.001) was associated with invasive MV. Coronary artery disease (33.3% vs. 7.1%; *p* = 0.002) and congestive heart failure (31.8% vs. 6.3%; *p* < 0.001) were associated with in-hospital death. Diabetes mellitus (OR, 2.43; 95% CI, 1.51–3.90; *p* < 0.001) and congestive heart failure (OR, 2.43; 95% CI, 1.06–5.87; *p* = 0.049) were independent predictors of in-hospital death [55]. Here we present the percentage of infected patients with the most common comorbidities: Hypertension (Figure 6), diabetes (Figure 7), cardiovascular disease (Figure 8).

There are some values that are clearly different from the majority. This may be caused by population heterogeneity, such as higher percentage of male population presented in the study [24,33,53,62], higher age [24,53,56], higher percentage of black race [46,56] or ICU sample [62]. In paper [56], the authors selected patients with and without CAD to compare these two groups. From six studies with the highest numbers, four were performed in the USA [33,46,56,62] and two in Italy [24,53]. In an article from Pakistan [14], the mean age of patients is lower than in the vast majority of other articles (44.6 ± 15.2).

There are some values that are clearly outstanding and, similar as in Figure 6, this may be caused by population heterogeneity, such as higher percentage of the male population presented in the study [33,62], higher age [56], the higher percentage of black race [46,56] or ICU sample [62]. In paper [56], the authors selected patients with and without CAD to compare these two groups. All five studies with the highest numbers were performed in the USA [33,45,46,56,62].

In paper [58], the percentage of patients with CAD is outstanding due to study design and chosen population sample: the authors compared SARS-CoV-2-infected patients with CAD to SARS-CoV-2-infected patients without CAD.

## 4. Discussion

This work focuses on cardiological complications caused by the SARS-CoV-2 virus. There is a possibility that its mechanism of interaction of cells and the human organism can be similar or approximate to other well-known viruses: influenza, SARS-CoV-1 or MERS-CoV [63]. Cardiac injury amongst COVID-19 patients is quite common, and the numbers oscillate with the range of 16.8–66.4% for myocardial injury in general (with no regards to injury time or past medical history) [7,8,9,10,11,12] and with a range of 2.9–10.8% for new-onset cardiac injury [10,17,18,19,20]. Those percentages are higher in patients with more severe/critical COVID-19 course [13,14,15,16,17,21], but we must remember that patients referred to intensive care wards usually have a more severe course of SARS-CoV-2 infection, as well as more risk factors such as age, male sex or comorbidities (hypertension or diabetes). Will it be temporary or permanent is a matter of research in the near future, but biomarkers of cardiac injury in SARS-CoV-2-infected patients usually decrease after a longer period of time, which can suggest direct cardiac damage caused by the virus [7]. However, it is known that there are various reasons for cardiac injury [64], and it can be caused by other viruses, for example, influenza virus [65,66]. Generally, infection increases the risk of cardiovascular events, such as acute myocardial infarction [67], and may deteriorate or trigger cardiovascular disease [68,69,70]. Nonetheless, there is a possibility that cardiovascular complications can be more severe for SARS-CoV-2 than other viruses, such as the influenza virus, mentioned before [63], and further research is needed to confirm this hypothesis. It is not a surprise that mortality is higher in patients with cardiac injury and/or elevated troponin levels comparing groups of COVID-19- positive patients with and without cardiac injury [7,9,10,15,16,32] but also those who are older [7,11,16,22,26], male [10,16,22] or have a more severe disease [7,22]. SARS-CoV-2-infected patients with more comorbidities are more likely to have cardiac injury [22,26]. The fact that cardiac injury [21,22], as well as elevated biomarkers [7,8,18,19,28,29,30,31,32], are an independent risk factor for mortality in patients infected with SARS-CoV-2 is crucial. One of the works showed that cTnI is a better short-term morality marker than LDH, CRP or D-Dimers [31]. Mortality (magnitude), admission time (time from symptom onset to first medical contact), frequency of complications and severity of cases increased during the COVID-19 pandemic compared to non-pandemic years [35,36,37], but this situation applies not only to the cardiological field but to every sector of the healthcare system, for example, presentation of patients with cancers and lower number of cancer-screening examinations [71].

Although virus genetics are found in heart tissue, myocarditis is not common [38,40,41], pericardial effusion occurred more often [21], but SARS-CoV-2, like every virus, has a multiplicitous possibility to interfere with living cells. Like SARS-CoV-1 [72], SARS-CoV-2 uses its SPIKE protein to bind to the ACE2-receptor, which can also be found on the surface of a host cell [63,73,74] and in heart tissue [75]. COVID-19 illness is likely to be accompanied by a hypercoagulable state [76] and microangiopathy [77]. Whether cardiac injury is caused by thromboembolism phenomena [7], direct damage [78] or inflammation factors [79] is still a matter of question and further research.

Combining general arrhythmias, only-AF (atrial fibrillation) and only-AA (atrial arrhythmia) studies, we can summarise and say that arrhythmias occur during COVID-19-related hospitalisation with a range of 7.2–26.5% of the patient population [31,32,33,34,36,37]. The most common are atrial arrhythmias, with the majority being AF. [42,44,46,47,48]. Brand-new arrhythmia occurs with a range of 5.3–13.3% of SARS-CoV2-infected patients [42,43,44,46,47,48,50]. Likewise, with cardiac injury, these patients are usually older and have more comorbidities than patients without arrhythmias [42,48,49,51], especially in the critical groups [13,42,53,54]. As we know, infections can be a cause of cardiac arrhythmias. It is not a rare sight when a patient with influenza virus, especially with more severe symptoms, develops atrial fibrillation [80], which seems to be one of the most common atrial arrhythmias. This applies not only to viral infections but pneumonia in general, which may initiate the phenomenon of arrhythmia in a patient [81] inter alia by triggering a reaction of the immune system, producing inflammation factors or increasing activation of the autonomic nervous system.

MRI is a very useful diagnostic device, and our research shows that abnormalities in the heart tissue were not common; however, the number of articles on this topic and patients who participated in such examinations were not sufficient [39].

The most common comorbidities in COVID-19-positive patients were hypertension (range:14.4–77%, median 47,8%) diabetes (range: 14–47%, median 23.7%) and cardiovascular disease (range: 8.7–56.7%, median 14.1%). The pathologies mentioned above were also popular amongst patients suffering from the MERS-CoV virus [82] and are possible to accompany every viral infection. Even if we are aware of the influence of viruses on our organisms, the SARS-CoV-2 virus seems to be unusually dangerous for the human population, and its long-term consequences are still uncertain.

## 5. Conclusions

Cardiac injury in the process of SARS-CoV-2 infection applies to approximately every fourth infected patient, whereas in nearly every tenth infected patient, it appears de novo. Myocarditis is not common and no different than in the healthy population. Nearly every seventh patient of the COVID-19-infected population has arrhythmias (which are mostly atrial), and almost every tenth patient has brand-new arrhythmias. The elevation of cardiac biomarkers (troponin, CK-MB, myoglobin) is an independent predictor of mortality in those patients. Comorbidities are crucial factors that worsen the outcome of a patient. Most common are hypertension, diabetes and cardiovascular disease. It is certain SARS-CoV-2 affects the cardiovascular system in many different ways and has an impact on the heart, electrical conduction system and vessels, which may suggest that we still need to continue research and expand our knowledge in order to better understand the virus itself.

## Figures and Tables

**Table 1:** Articles included in the study.

Number	Country	Publication Date	Number of Citations	Study Time	Sample Size	Median Age (Years)	Mean Age (Years)	Males
7	China	24.02.21	1	01.01.20–27.02.2020	181	55 (46–65)		56.4%
8	US	15.02.21	4	01.03.20–16.04.2020	2895	–	–	–
9	Italy/USA	30.01.21	2	21.02.20–31.05.2020	313	66.1 (55.1–79.4)		56.2%
10	UK/Spain	08.03.21	1	Second half of March 2020	434	66 (56–80)		62.9%
11	Turkey	25.03.21	0	15.04.20–death/discharge	324		55.1 ± 12.9	52.8%
12	USA	13.08.21	0	04.04.20–05.06.2020	270	70 (57–78)		51.1%
13	Pakistan	01.03.21	5	01.04.20–31.08.2020	201		44.6 ± 15.2	59.2%
14	China/USA	12.02.21	2	01.02.20–10.03.21	330	67 (range: 42–92)		48.8%
15	USA	13.11.20	23	15.03.20–11.06.20	243		62.8 ± 14.9	60.9%
16	France	02.03.21	2	26.02.20–20.04.20	2878		66.6 ± 17.0	57.9%
17	China/USA	28.01.21	9	15.03.20–01.04.2020	218	62 (55–69)		49.6%
18	USA	19.01.21	3	10.03.20–05.08.2020	179		59.8 ± 16.9	62%
19	UK	20.02.21	3	03.03.20–21.04.2020	176		55.1 ± 12.9	71%
20	USA	10.05.21	3	09.07.20–21.10.20	137	20 (range: 18–27)		68%
21	China	18.01.21	6	11.03.20–16.03.2020	132		55.1 ± 12.9	51.5%
22	China/USA/UK	19.02.21	1	11.01.20–25.03.2020	304	65 (54.0–74.0)		52.6%
23	China	09.02.21	5	29.01.20–04.03.2020	128		61.3 ± 13.1	47.7%
24	Italy/Switzerland	20.07.21	3	01.03.20–22.04.20	226		68.9 ± 13.9	62.4%
25	USA	16.04.21	1	till 13.07.2020	367		61 ± 17	60%
26	China	11.01.02	1	03.01.20–26.02.2020	400	49 (37–61)		52.2%
27	China	29.01.21	0	27.01.20–12.02.20	726	68 (58–77); 65 (55–71) *		54.1%
28	Spain	16.02.21	3	01.03.20–30.03.20	2873	66 (54–76)		59.1%
29	China	05.03.21	1	01.01.20–28.02.20	242	68 (61–75)		62.4%
30	China	18.02.21	1	20.01.20–10.04.20	173	73.0 (64.0–80.5)		64.2%
31	Spain	18.01.21	4	16.03.20–15.05.20	196	67.5 (53.5–78.0)		59.7%
32	Israel	26.02.21	4	09.02.20–28.08.2020	320	73.31 (61.33–82.25); 59.83 (49.65–71.19) **		64.1%
33	USA	10.11.20	10	01.03.20–01.04.20	887		64 ± 17	58%
34	UK	27.01.21	3	01.03.20–15.04.2020	346	65 (59–74) 74 (63–83) ***		64.8%; 43.7% ♥
35	Germany	06.12.20	11	01.01.20–30.04.2020	147	70 (56.5–76); 64 (58–72) ♥♥		69%; 70% ♥♥♥
36	Spain	17.03.21	2	01.03.20–30.04.2020	316	68 (58.8–78); 66 (56–77) ♦		71.2%; 70.3% ♦♦
37	Japan	05.02.21	8	01.01.18–06.04.20 || 07.04.20–14.08.2020	422	72(61–80); 70 (59–79) ♦♦♦		72.1%; 66.7% ♣
38	Italy	26.02.21	6	04.03.20–20.05.2020	1169		70.96 ± 16.71	48%
39	UK/USA	18.02.21	53	01.01.2020–31.10.20	148		64 ± 12; 64 ± 9 ♣♣	56 %; 88 % ♣♣♣
40	USA	04.03.21	34	05.2020–10.2020	789		25 ± 3	98.5%
41	UK	17.12.20	35	–	146	20 (19–21); 30 (27–34); 25 (22–27) ♠		37%; 63%; 88% ♠♠
42	Israel	30.10.20	5	01.02.20–30.05.20	390	57.5 (43–74.3)		55.4%
43	Portugal	08.2021	0	12.04.20–10.05.20	692	73.5 (61–80.25) ♠♠♠		70.3% ☼
44	Germany	02.01.21	11	05.03.20–17.06.20	166		73.6 ± 12.8; 61.6 ± 16.7 ☼☼	67.6%; 64.6% ☼☼☼
45	USA/India	19.03.21	5	04.2020–06.2020	186	60 (range: 18–95).		53.2%
46	USA	16.04.21	1	01.03.20–01.05.20	310		61.4 ± 16.	41.3%
47	USA	22.01.21	12	01.03.20–27.04.20	9564		64.8 ± 16.	58.9%
48	USA	20.02.21	0	01.03.20–15.04.20	1029		63.6 ± 17.4	57%; 56% §
49	Spain	28.01.21	1	10.03.20–15.04.20	517		68.1 ± 15.1	
50	Denmark	13.03.21	3	01.03.20–01.06.20	155	66 (55–74)		73%
51	Spain	03.11.20	11	03.2020–04.2020	160		75.9 ± 9.6; 64.9 ± 16.3 §§	66.7%; 59.5% §§§
52	China	24.01.21	0	01.02.20–19.03.20	463	61 (51–69)		47.9%
53	Italy	29.01.21	5	01.03.20–10.04.20	216	67.0 (56.75–79.0)		66%
54	China	12.03.21	1	24.02.20–05.04.20	168		61.60 ± 11.30; 61.90 ± 13.90 •	44%; 52.4% ••
55	Korea	29.12.20	4	15.02.20–24.04.20	2269		55.5 ± 20.2	35.9%
56	USA	01.08.21	8	01.03.20–24.04.20	355		66.21 ± 14.21	49%
57	USA	09.01.21	20	01.04.20–30.09.20	8383		71.7 ± 13.2	49.8%
58	China	16.03.21	4	12.02.20–16.03.20	157		62 ± 13	50.3%
59	China	30.01.21	3	01.02.20–26.02.20	148		57.2 ± 17.7	45.3%
60	USA	02.02.21	0	–	204		64 ± 13	76%
61	USA/Israel	25.01.21	5	01.01.20–08.06.20	589		66 ± 18	56%
62	USA	31.03.21	1	29.02.20–28.06.20	300		60 ± 16	60%

* Critical patients: 68 (58–77); Severe patients: 65 (55–71). ** Elevated troponin: 73.31 (61.33–82.25); Non-elevated troponin: 59.83 (49.65–71.19). *** COVID-19 patients: 65 (59–74); Non-COVID-19 patients: 74 (63–83). ♥ COVID-19 patients: 64.8%; Non-COVID-19 patients: 43.7%. ♥♥ pre-COVID-19 patients: 70 (56.5–76); COVID-19 patients: 64 (58–72). ♥♥♥ pre-COVID-19 patients: 69%; COVID-19 patients: 70%. ♦ pre-COVID-19 patients: 68 (58.8–78); COVID-19 patients: 66 (56–77). ♦♦ pre-COVID-19 patients: 71.2%; COVID-19 patients: 70.3%. ♦♦♦ pre-COVID-19 patients: 72 (61–80); COVID-19 patients: 70 (59–79). ♣ pre-COVID-19 patients: 72.1%; COVID-19 patients: 66.7%. ♣♣ COVID-19 patients: 64 ± 12; Healthy control: 64 ± 9. ♣♣♣ COVID-19 patients: 56 %; Healthy Control: 88%. ♠ COVID-19 athletes: 20 (19–21); Healthy control: 30 (27–34); Athlete control: 25 (22–27). ♠♠ COVID-19 athletes: 37%; Healthy control: 63%; Athlete control: 88%. ♠♠♠ Patients with arrhythmias: 73.5% (61–80.25). **☼** Patients with arrhythmias: 70.3%. **☼☼** Patients with arrhythmias: 73.6 ± 12.8; Patients without arrhythmias 61.6 ± 16.7. **☼☼☼** Patients with arrhythmias: 67.6%; Patients without arrhythmias: 64.6%. **§** Atrial arrhythmias patients: 57%; No-atrial arrhythmias group: 56%. **§§** Patients with atrial fibrillation: 75.9 ± 9.6; Patients without atrial fibrillation: 64.9 ± 16.3. **§§§** Patients with atrial fibrillation: 66.7%; Patients without atrial fibrillation: 59.5%. • Patients with bacterial inflammation: 61.60 ± 11.30; Patients with virus inflammation: 61.90 ± 13.90. •• Patients with bacterial inflammation: 44%; Patients with virus inflammation: 52.4%.

## Abbreviations

AA—atrial arrhythmias, ACS—acute cardiac syndrome, AF—atrial fibrillation, BMI—body mass index, CAD—cardiovascular artery disease, CKD—chronic kidney disease, CK-MB—creatine kinase heart protein variant, CMR—cardiac magnetic resonance, COVID-19 disease—patients infected with SARS-CoV-2 virus, D-Dimers—fibrin degradation product, ECG—electrocardiography, HF—heart failure, HFrEF—heart failure with reduced ejection fraction, ICU—intensive care unit, MI—myocardial injury, NT-proBNP—brain natriuretic peptide, TTE—trans-thoracic echocardiogram, cTn—cardiac troponin, hs-cTn—high sensitivity cardiac troponin, cTnI—cardiac troponin type I, cTnC—cardiac troponin type C, cTnT—cardiac troponin type T, ULN—upper limit of normal.
